# Isavuconazole as an optimal treatment option for multiple pathogens induced severe pneumonia in immunocompromised hosts: a case series report

**DOI:** 10.3389/fmed.2025.1565071

**Published:** 2025-04-28

**Authors:** Xinyin Wang, Yanjing You, Shuyang Chen, Peiyu Wang, Shengyuan Zeng, Liying Zhuang, Meng Wang, Guoxiang Lai, Zongyang Yu, Guoqing Yu, Wen Wen

**Affiliations:** ^1^Department of Pulmonary and Critical Care Medicine, Fuzong Clinical Medical College of Fujian Medical University, Dongfang Hospital of Xiamen University, School of Medicine, Xiamen University, 900th Hospital of PLA Joint Logistic Support Force, Fuzhou, Fujian, China; ^2^Department of Pulmonary and Critical Care Medicine, Zhongshan Hospital, Fudan University, Shanghai, China; ^3^Department of Nephrology, Fuzong Clinical Medical College of Fujian Medical University, Dongfang Hospital of Xiamen University, School of Medicine, Xiamen University, 900th Hospital of PLA Joint Logistic Support Force, Fuzhou, Fujian, China

**Keywords:** severe pneumonia, immunocompromised hosts, invasive aspergillosis, isavuconazole, antifungal therapy

## Abstract

**Background:**

Treating severe pneumonia caused by multiple pathogens in immunocompromised hosts (ICHs) presents significant challenges. Isavuconazole (ISA), a next-generation triazole antifungal agent, has shown promise in managing fungal infections. However, clinical evidence regarding its efficacy in cases of complex infections involving multiple pathogens in ICHs remains limited.

**Case presentation:**

This study describes a case series of three ICHs diagnosed with severe pneumonia, including invasive aspergillosis (IA). All three patients received ISA-based personalized antimicrobial regimens. Alleviation of symptoms was observed in all patients following antimicrobial treatment, with notable absorption of pulmonary lesions and no significant hepatorenal toxic side effects, with no recurrence observed.

**Conclusion:**

ICHs are highly susceptible to fungal infections, and the severity of their condition can escalate dramatically, with a significant risk of mortality, when severe pneumonia caused by multiple pathogens occurs concurrently. A stepwise treatment strategy, which balances the use between immunosuppressant and effective antimicrobial treatment, is crucial. The selection of appropriate drugs should account for potential adverse drug reactions (ADRs). In this case series, ISA exhibited robust efficacy in treating IA with minimal ADRs. Therefore, ISA represents a valuable option for managing severe pneumonia in ICHs, particularly in the context of IA and co-infections caused by multiple pathogens.

## Highlights

• ICHs are susceptible to severe pneumonia caused by IA with high mortality.
• When ICHs are infected with severe pneumonia caused by IA and other pathogens, a stepwise treatment strategy is essential.
• ISA is the next-generation triazole agent with few contraindications, minimal ADRs, proven efficacy, and economic benefits.
• ISA is an effective treatment option for severe pneumonia caused by complex infections including IA in ICHs.

## Introduction

Recent epidemiological studies indicate that the use of advanced medical interventions, such as organ transplantation, cytotoxic therapies, and widespread use of immunosuppressive medications, have significantly increased the population of ICHs (1). ICHs are susceptible to opportunistic infections, including *Aspergillus*, as well as the reactivation of latent pathogens like *Pneumocystis jirovecii* (PJ) and herpesviruses (2). Such infections in ICHs can lead to severe pneumonia and even acute respiratory distress syndrome (ARDS), necessitating intensive care unit (ICU) admission (3, 4).

Severe pulmonary infections in ICHs, particularly those involving invasive fungal pathogens, are associated with alarmingly high mortality rates (5). Management of these patients poses significant challenges due to the delicate immune balance which complicates the choice of treatment strategies. Therefore, optimizing therapeutic approaches for ICHs with complex infections, including *Aspergillus*, is crucial for reducing hospital stays, improving survival rates, and enhancing prognosis. ISA, a novel triazole antifungal agent (marketed as Cresemba^®^), serves as a pivotal therapeutic option for severe pneumonia secondary to invasive fungal infections, particularly when voriconazole (VOR) or posaconazole are contraindicated owing to toxicity, unfavorable pharmacokinetics, or drug interactions (6).

Herein, we report three cases of severe pneumonia involving IA and co-infections with multiple pathogens. All three cases were successfully treated using ISA in combination with other antimicrobial agents.

## Materials and method

This report provides the diagnosis and treatment process of three patients with severe pneumonia caused by *Aspergillus* and other multiple pathogens. These patients were treated from March to August 2023 at the Respiratory and Critical Care Medicine Department of the 900th Hospital of the Joint Logistics Support Force, PLA. The analysis includes their clinical characteristics, treatment plans, prognosis, and outcomes.

### Case description

#### Case 1

**FIGURE 1:**
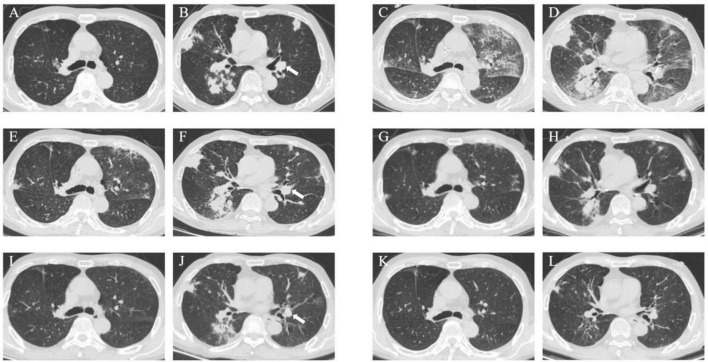
Case 1, Chest CT scans. **(A, B)** On March 30: multiple ill-defined pulmonary nodules in both lungs. The largest areas of consolidation appearing with inhomogeneous density. In the left lower lobe, diagonal measurement result was 3.5 × 4.4 cm (arrow). **(C, D)** On April 7: patchy opacities and ground-glass attenuation in both lungs. **(E, F)** On April 14: partial resolution of the inflammation. The largest consolidated nodules enlarged to 4.8 × 6.2 cm (arrow). **(G, H)** On April 27: multiple patchy opacities of the left lung were further absorbed than that on April 14, while opacities of the right lung were similar to before. **(I, J)** On June 26: the pulmonary nodules and patchy opacities were gradually resolving after systemic therapy. The largest consolidation was about 4.0 × 3.3 cm (arrow). **(K, L)** On August 30: the pulmonary nodules and patchy opacities gradually diminished.

A 73-year-old male was hospitalized on 30 March 2023, with a two-month history of a productive cough and shortness of breath, which had worsened over the past week. A previous chest CT scan from another hospital revealed bronchiectasis and a suspected infection in the upper lobe of the right lung. The patient had experienced a weight loss of 5 Kg in the preceding week. Upon admission, a chest CT scan revealed multiple ill-defined pulmonary nodules in both lungs, with the largest area of consolidation (measuring 3.5 cm × 4.4 cm) located in the left lower lobe, characterized by inhomogeneous density (arrow) (Figures 1A, B).

The patient had a 30-year smoking history, a one-month history of hypertension, and a diagnosis of membranous nephropathy two months earlier. He was on a regular regimen of tacrolimus and methylprednisolone (MP) for the membranous nephropathy (specific dosing details unspecified). The results of physical and laboratory tests upon admission were summarized in Table 1, indicated systemic infection, respiratory failure, and hepatic and renal dysfunction. Additionally, arterial blood gas analysis and tests for cytomegalovirus (CMV) DNA and galactomannan (GM) were positive. Cardiac markers, including myoglobin (> 1,000 μg/L), ultra-sensitive troponin I (0.191 μg/L), and creatine kinase MB mass (25.82 μg/L), were elevated.

**TABLE 1:** Patients’ basic conditions and results of physical / laboratory tests.

Measure	Patient 1 on admission	Patient 2 on admission	Patient 3 on admission	Reference range
Age (year)/sex	73/M	44/F	52/M	
Symptoms	Sputum-producing cough; breathlessness	Recurrent fever; breathlessness	Recurring fever; breathlessness; chest pain	
Underlying condition	Membranous nephropathy; hypertension	Bilateral uveitis; hypertension	Post-allogeneic kidney transplantation; a left thyroidectomy; hepatitis B; hypertension	
Pre-admission immunosuppressant use	Tacrolimus (details unspecified)	MMF 6 pills qd; methotrexate 12.5 mg qw; adalimumab 400 mg q2w	Tacrolimus 1 g bid; sirolimus 1 tablet qd	
Pre-admission steroid use	MP (details unspecified)	MP 16 mg qd	Prednisone 2 tablets qd	
Body temperature (°C)	36.5	36.2	36	36.1–37.2
Respiratory rate (breaths/min)	18	18	16	12–20
Heart rate (beats/min)	58	101	102	60–100
Blood pressure (mmHg)	154/85	105/77	141/95	90/60–120/80
pH	7.43	7.45	7.33	7.35–7.45
HCO_3_ (mmol/L)	19	16.7	12	22–27
PO_2_ (mmHg)	59	59	112	80–100
PCO_2_ (mmHg)	27	24	23.3	35–45
SaO_2_ (%)	91.7	95.6	97	95–98
FiO_2_ (%)	29	50	33	21–100
OI (mmHg)	203	118	339	> 300
WBC count (10^9^/L)	6.38	5.29	7.31	3.5–9.5
Neutrophil (%)	92.9	87.1	81.7	40–75
Granulocyte Count (10^9^/L)	5.88	4.6	5.97	1.8–6.3
Lymphocyte count (10^9^/L)	0.24	0.45	0.8	1.1–3.2
CRP (mg/L)	105	83.7	148	0–10
PCT (ng/mL)	1.08	0.08	10.9	< 0.05
D-Dimer (mg/L)	0.72	11.14	2.68	0–0.5
ESR (mm/h)	97	98	120	M: 0–15 F: 0–20
Urea (mmol/L)	19.9	6.2	20.9	2.9–8.2
Creatinine (μmol/L)	118.4	81.8	458	53–115
Uric acid (μmol/L)	589.6	133.5	605.1	155–428
LDH (U/L)	297.1	1,180.1	780.2	109–245
Albumin (g/L)	21.7	33.3	33.2	40–55
ALT (U/L)	32	168.1	17.4	7–50
AST (U/L)	20.7	83.7	30.6	13–40
Cytomegalovirus DNA	Positive	Positive	Negative	Positive: > 5.82 × 10^4^ copies/m
GM test	Positive	Negative	Positive	
G test	NA	NA	Negative	
Tests for respiratory pathogen antibodies	Negative	Negative	Negative	
Mycoplasma serological testing	Negative	Negative	NA	
Tuberculosis antibody testing	Negative	Negative	Negative	
IGRA	Negative	Negative	Negative	
TST	Negative	Negative	Negative	
Sputum smear	Negative	Negative	Negative	
Sputum culture	Negative	Negative	Negative	
BALF culture	*Aspergillus flavus*; *Nocardia*; Pseudodiphtheritic bacillus	*Klebsiella pneumoniae*; Penicillium	*Aspergillus* niger	
BALF GM test	Positive	Positive	Negative	
BALF tuberculosis DNA	Negative	Negative	Negative	

M, male; F, female; NA, not obtained; PO_2_, partial pressure of oxygen; PCO_2_, partial pressure of carbon dioxide; SaO_2_, oxygen saturation in arterial blood; FiO_2_, fraction of inspired oxygen; OI, oxygenation index; WBC, white blood cell; CRP, C-reactive protein; PCT, procalcitonin; ESR, erythrocyte sedimentation rate; LDH, lactate dehydrogenase; ALT, alanine aminotransferase; AST, aspartate aminotransferase; GM test, galactomannan test; G test, (1, 3)-β-D-glucan test; IGRA, interferon-gamma release assay; TST, tuberculin skin test; BALF: bronchoalveolar lavage fluid; MMF, mycophenolate mofetil; MP, methylprednisolone.

On March 31, a fiberoptic bronchoscopy was performed, and bronchoalveolar lavage fluid (BALF) was collected for metagenomic next-generation sequencing (mNGS). The mNGS results were summarized in Table 2. While BALF culture results identified the presence of *Aspergillus flavus*, *Nocardia*, and Pseudo-diphtheritic bacillus. Additionally, the BALF GM test returned positive, while the tuberculosis DNA test was negative. Based on clinical symptoms, laboratory tests, and imaging findings, the patient was diagnosed with severe pneumonia and type I respiratory failure, attributed to a complex infection involving at least six pathogens, including PJ, *Nocardia*, *Aspergillus* (*fumigatus* and *flavus*), nontuberculous mycobacteria (NTM), CMV, *Klebsiella aerogenes*, yeasts, and bacilli.

**TABLE 2:** mNGS results of Case 1.

Microbe	Microbial reads	Relative abundance
*Pneumocystis jirovecii* (PJ)	38,413	15.52%
*Nocardia arthritidis*	3,768	1.53%
Nontuberculous mycobacteria (NTM)	453	0.19%
*Aspergillus fumigatus*	421	0.18%
*Candida albicans*	304	0.13%
*Candida glabrata*	242	0.10%
*Klebsiella oxytoca*	295	0.12%
Cytomegalovirus (CMV)	12	< 0.01%
Others	20,349,980	82.22%

Presented as microbial reads and relative abundance.

Concurrent administration of multiple anti-infective agents (antibacterial, antifungal, antiviral, and anti-NTM therapies) posed a high risk of drug interactions, hepatorenal strain, and adverse effects such as gastrointestinal intolerance. Therefore, treatment was implemented in two phases, prioritizing rapidly progressing pathogens with high mortality risks (PJ, *Nocardia*, *Klebsiella aerogenes*, and CMV) in Phase 1 and addressing slow-growing pathogens (e.g., *Aspergillus* and NTM) in Phase 2.

##### Phase 1: Initial management

**FIGURE 2:**
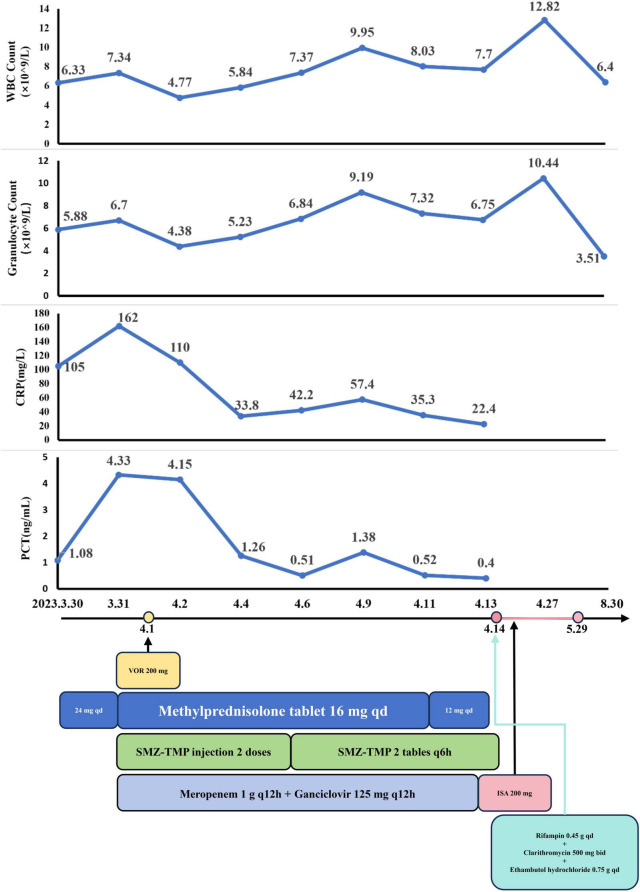
The dynamic of laboratory indicators and treatment timeline for case 1. MP, Methylprednisolone; SMZ-TMP, Sulfamethoxazole and Trimethoprim; VOR, Voriconazole; ISA, Isavuconazole.

On March 30, the patient continued with 24 mg of MP daily (Figure 2). Starting March 31, sulfamethoxazole-trimethoprim (SMX-TMP) injections were administered every 8 h to target *Nocardia* and PJ. After six days, due to adequate blood drug levels, the regimen was adjusted to 160 mg every six hours. Meropenem (1 g every 12 h) and ganciclovir (125 mg every 12 h) were prescribed for antibacterial and antiviral purposes, respectively. By April 4, inflammatory markers (CRP: 33.8 mg/L; PCT: 1.26 ng/mL) had decreased, reflecting slight clinical improvement. However, a chest CT on April 7 revealed progression of pneumonia (Figures 1C, D). Despite this, the treatment plan was maintained due to the delayed response in imaging. MP remained the sole immunosuppressive drug.

##### Phase 2: Antifungal and anti-NTM therapy

By April 13, there was a significant improvement in the patient’s symptoms, evidenced by the reduction in cough, sputum production, and wheezing. Arterial blood gas analysis showed normalization [FiO_2_: 43%, pH: 7.54, PO_2_: 125 mmHg, PCO_2_: 27 mmHg, ${\text{HCO}}_{3}^{-}$: 26 mmol/L, SaO_2_: 99.4%, oxygenation index (OI): 291 mmHg], and inflammatory markers (CRP: 22.4 mg/L; PCT: 0.40 ng/mL) continued to decline. CMV DNA tests were negative twice consecutively. On April 14, partial resolution of pulmonary inflammation was observed via CT, although the largest consolidated nodule had increased to 4.8 cm × 6.2 cm (arrow) (Figures 1E, F). Meropenem and ganciclovir were discontinued.

VOR (April 1–April 13) was initially used for antifungal treatment but was replaced by ISA on April 14, due to suboptimal blood levels resulting from rapid drug metabolism. ISA was administered as 200 mg every 8 h for the first 48 h, followed by 200 mg daily. Additional treatments included SMX-TMP (21 days), rifampin (0.45 g qd), clarithromycin (500 mg bid), and ethambutol hydrochloride (0.75 g qd) for NTM. On April 17, the BALF fungal culture revealed no growth. By April 27, the left lung opacities showed further resolution, while the right lung opacities remained stable (Figures 1G, H).

Despite financial constraints leading to ISA (April 14–May 29) discontinuation after 45 days, follow-up on June 26 showed further reduction in pulmonary lesions. The largest consolidation had decreased to 4.0 cm × 3.3 cm (arrow) (Figures 1I, J). By August 30, pulmonary nodules and patchy opacities had gradually diminished.

#### Case 2

A 44-year-old female was admitted to the hospital on 25 June 2023, presenting with a 20-day history of recurrent fever and three days of breathlessness. Her fever peaked at 40°C, accompanied by chills, light-headedness, and exhaustion. After self-medicating without improvement, she sought treatment at a local hospital. A chest-enhanced CT scan revealed diffuse inflammation in both lungs, bronchiectasis in the right middle lobe, lingula of the left upper lobe, and the medial basal segment of the right lower lobe, as well as situs inversus totalis. The patient tested positive for COVID-19.

BALF mNGS results identified multiple pathogens, including PJ, *Aspergillus fumigatus*, *Marnier-Lapostolle blue bacteria*, CMV, COVID-19, *Klebsiella pneumoniae*, and *Acinetobacter baumannii*. Initial treatment at the local hospital included cephalosporins for bacterial infections, “Simnotrelvir/Ritonavir” for viral infections, “sulfamethoxazole” for PJ, and VOR for fungal infections. Despite these interventions, her symptoms did not improve, and follow-up testing showed abnormal liver function. She was subsequently transferred to our hospital.

**FIGURE 3:**
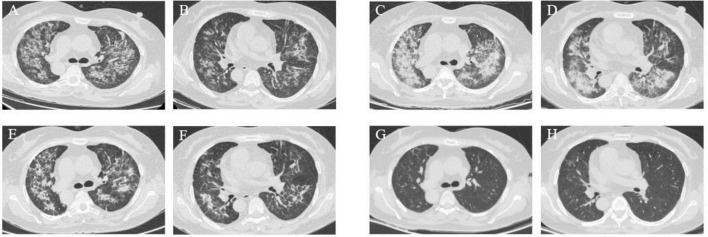
Case 2, chest CT scans. This patient had situs inversus totalis. **(A,B)** On June 26: bronchial dilation in the upper left lung and middle right lung. Multiple patchy opacities and ground-glass attenuation in both lungs. **(C,D)** On June 30: significant progression of pneumonia. **(E,F)** On July 6: partial resolution of the patchy opacities and ground-glass attenuation. **(G,H)** On August 28: significant improvement and absorption of patchy opacities were observed after the systemic antifungal therapy.

On June 26, chest CT scans confirmed the findings of situs inversus totalis, along with bronchial dilation in the upper left and middle right lungs. Multiple patchy opacities and ground-glass attenuation were observed in both lungs (Figures 3A, B). The patient had been diagnosed with bilateral uveitis one year earlier and is on a comprehensive treatment regimen, including MP 16 mg qd, mycophenolate mofetil (MMF) 6 pills qd, methotrexate 12.5 mg qw, and adalimumab 400 mg q2w (Table 1). Besides, the patient had a one-year history of hypertension. Physical examination revealed coarse breathing sounds in both lungs. BALF mNGS results identified several pathogens, including PJ (*n* = 49,518 reads, 33.20%), *Klebsiella pneumoniae* (*n* = 181 reads, 0.13%), *Aspergillus fumigatus* (*n* = 82 reads, 0.06%), CMV (*n* = 8,292 reads, 5.56%), and others (*n* = 91,050 reads, 61.05%). The GM test was positive, while the tuberculosis DNA test was negative. Cultures from BALF grew *Klebsiella pneumoniae* and Penicillium. Based on these findings, the patient was diagnosed with severe pneumonia and type I respiratory failure caused by invasive pulmonary aspergillosis (IPA) and concurrent infections, including PJ, CMV, and *Klebsiella pneumoniae*.

##### Phase 1: Initial management

Given the patient’s abnormal liver function and the risk of hepatotoxicity from VOR, treatment was initiated with liver-protective measures. For PJ, SMX-TMP was administered as two injections q6h for three days, then transitioned to four tablets q6h. Meropenem (50 mL q8h) was prescribed for bacterial infections, ganciclovir (330 mg q12h) for CMV, and MP (16 mg qd) to prevent immunological rejection.

After three days, the patient showed significant clinical improvement, with resolution of fever and wheezing. Blood gas values were as follows: FiO_2_ 50%, pH 7.47, PO_2_ 81 mmHg, PCO_2_ 25 mmHg, ${\text{HCO}}_{3}^{-}$ 22 mmol/L, SaO_2_ 98.7%, and OI 162 mmHg. CRP levels decreased to 50 mg/L. However, follow-up chest CT scans showed progression of pneumonia (Figures 3C, D). Due to the delayed response in imaging, the initial treatment regimen was maintained.

##### Phase 2: Antifungal and comprehensive therapy

On June 29, treatment for aspergillosis was initiated with ISA at 200 mg q8h for the first 48 h, followed by 200 mg qd. To better control immunosuppression, MP was replaced with intravenous methylprednisolone sodium succinate (MPS) at 40 mg qd. By July 6, CT scans showed partial resolution of patchy opacities and ground-glass attenuation (Figures 3E, F), and liver function markers (AST, ALT) and CRP levels had normalized.

**FIGURE 4:**
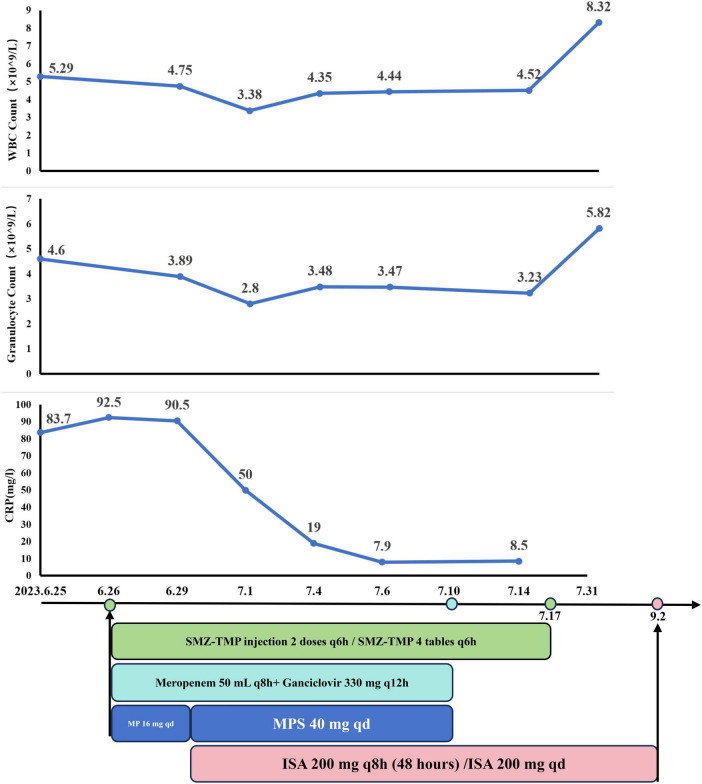
The dynamic of laboratory indicators and treatment timeline for case 2. SMZ-TMP, Sulfamethoxazole and Trimethoprim; MP, Methylprednisolone; MPS, methylprednisolone sodium succinate; ISA, Isavuconazole.

The patient was discharged on July 10 after completing a 14-day course of ganciclovir and meropenem. SMX-TMP was continued at four tablets q6h for 21 days. GM test results of July 14 and August 2 are negative. Follow-up CT scans on August 28 showed significant improvement, with further absorption of patchy opacities (Figures 3G, H). On September 2, ISA (June 29–September 2) was discontinued due to financial constraints, completing a 66-day treatment course (Figure 4).

#### Case 3

**FIGURE 5:**
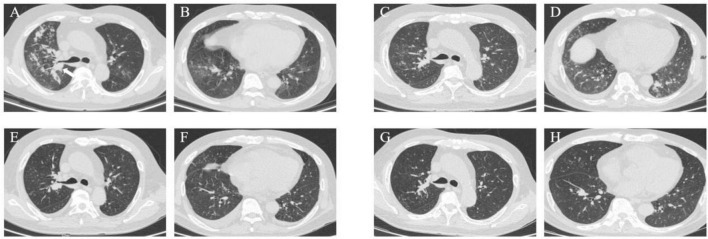
Case 3, chest CT scans. **(A, B)** On August 9: multiple patchy opacities and nodules in both lungs. The largest nodule was 1.8 × 1.7 cm in the basal segment of the right inferior lobe (arrow). **(C,D)** On August 17: slight improvement of the right pulmonary patchy opacities while progression of the lower lobe of the left lung. **(E,F)** On September 11: notable diminishment of multiple patchy opacities in both lungs. **(G,H)** On November 30: marked absorption of patchy opacities.

A 52-year-old male was admitted to our department on 9 August 2023, with a three-week history of recurrent fever, chest pain, and breathlessness. His temperature had peaked at 39°C, accompanied by chills and fatigue. A chest CT revealed multiple patchy opacities and nodules in both lungs. The largest nodule was 1.8 cm × 1.7 cm located in the basal segment of the right inferior lobe (arrow) (Figures 5A, B).

The patient received a kidney transplantation 18 years ago and is thus on long-term immunosuppressive medication, including tacrolimus (1 g bid), sirolimus (1 tablet qd), and prednisone (2 tablets qd). He had undergone a left thyroidectomy 11 years ago for thyroid cancer and had a 10-year history of hepatitis B managed with Entecavir, as well as a 4-year history of hypertension managed with Losartan. Following the recurrence and metastasis of thyroid cancer two years ago, the patient had been on regular treatment with Sorafenib. Physical examination upon admission revealed coarse breathing sounds, wet rales, and wheezing in both lungs. BALF mNGS analysis revealed PJ (*n* = 27,865 reads, 14.40%) and *Aspergillus fumigatus* (*n* = 54,451 reads, 28.13%). Laboratory and BALF mNGS findings, along with a positive influenza A RNA test from a throat swab, supported a diagnosis of IPA with concurrent infections from PJ and influenza A virus. Detailed laboratory test results are provided in Table 1.

##### Phase 1: Initial management

The initial treatment regimen targeted PJ with SMZ-TMP (160 mg q8h) for 21 days. Antiviral therapy included oseltamivir at 30 mg qd for 3 days, and cefoperazone-sulbactam (1.5 g q12h) was administered for antibacterial prophylaxis. Corticosteroids were prescribed to mitigate immunological rejection and inflammation.

A follow-up chest CT on August 17 revealed slight improvement in the right lung but progression in the lower lobe of the left lung (Figures 5C, D). This indicated ongoing pneumonia progression, prompting continuation of the original treatment plan.

##### Phase 2: Antifungal therapy and liver function management

The patient was discharged on August 28 and initiated antifungal treatment on VOR at 200 mg q12h. A follow-up chest CT on September 11 showed significant resolution of multiple patchy opacities in both lungs (Figures 5E, F). However, subsequent tests revealed a positive blood GM test and elevated liver enzymes (ALT: 212.4 U/L, AST: 202.3 U/L), leading to a diagnosis of drug-induced liver injury.

**FIGURE 6:**
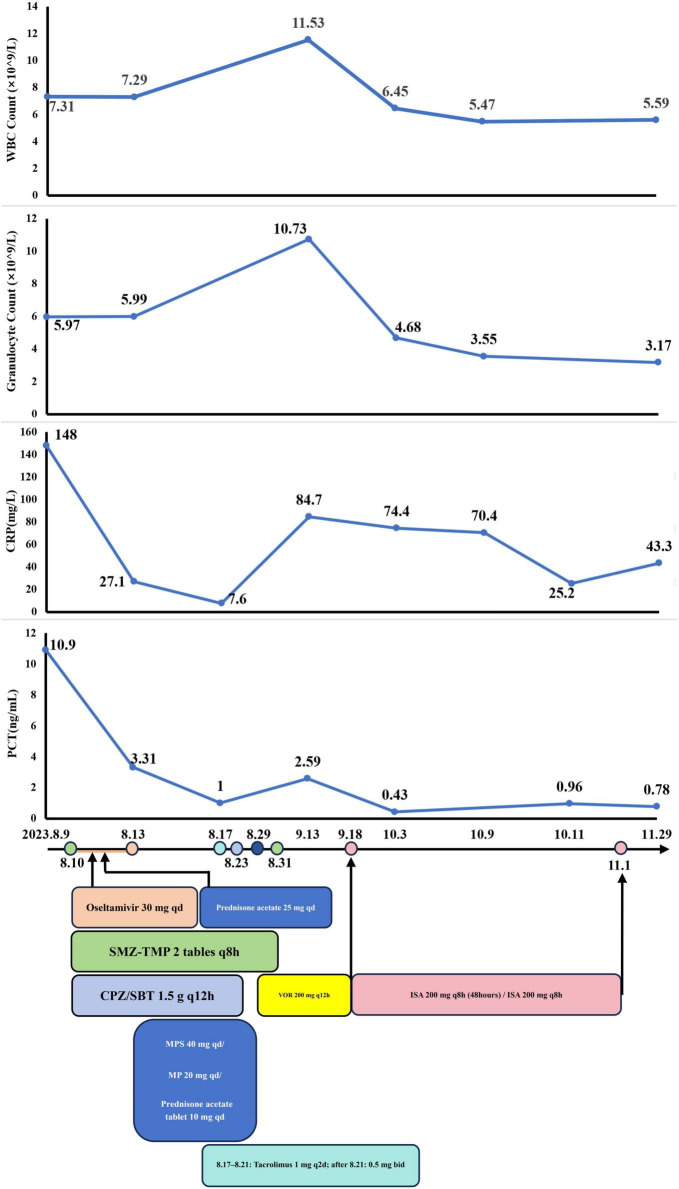
The dynamic of laboratory indicators and treatment timeline for case 3. SMZ-TMP, Sulfamethoxazole and Trimethoprim; CPZ/SBT, Cefoperazone Sulbactam; MP, Methylprednisolone; MPS, methylprednisolone sodium succinate; VOR, Voriconazole; ISA, Isavuconazole.

Consequently, on September 18, after completing a 21-day course of VOR (August 29–September 18), the anti-fungal medication was switched to ISA. By October 3, follow-up laboratory tests showed normalization of liver function, including ALT, AST, and complete blood count. ISA (September 18–November 1) treatment continued for 45 days (Figure 6) but was discontinued by the patient on his own initiative due to financial constraints.

Aside from the unscheduled review in November for personal reasons, the patient’s monthly routine evaluations of BALF cultures and GM test results from October 2023 to May 2024 were consistently negative. A chest CT on November 30 revealed marked absorption of patchy opacities (Figures 5G, H). The patient achieved substantial clinical and radiological improvement following antifungal therapy, particularly with ISA.

## Discussion

The 2022 WHO fungal priority pathogens list identifies *Aspergillus* as one of the four critical pathogens responsible for invasive fungal infections (7). Clinical presentations of IPA include breathlessness, fever, cough, chest or pleuritic pain, and hemoptysis (8). However, the classic halo sign (a pulmonary nodule surrounded by a region of low attenuation) is often absent in high-risk IPA patients, making radiological findings indistinguishable from other pulmonary infections (9). Due to severe immunodeficiency, ICHs infected with *Aspergillus* are more likely to develop IPA rather than noninvasive diseases (10).

ICHs include individuals with Human Immunodeficiency Virus (HIV), solid organ transplant recipients, cancer patients, and those undergoing immunomodulatory therapy for autoimmune diseases (11). IPA in ICHs is associated with high mortality and poses significant treatment challenges (12, 13). Recent studies report mortality rate of 40%–50% in *Aspergillus* infections among patients with acute leukemia and hematopoietic stem cell transplants (14–16). A 2021 review by Paramythiotou et al. (17) evaluated six ICU patients with COVID-19-associated IPA and no prior history of immunosuppression. Five patients were treated with ISA and one with another antifungal medication, resulting in a 67% mortality rate (4/6). Among those treated with ISA, two were transferred to a general ward, while the other three died (17). Nevertheless, reports of successful ISA use in ICHs with IPA and co-infections remain limited. In our study, we present three cases of ICHs with IPA and co-infections causing severe pneumonia and respiratory failure. Combination therapies, including ISA and other medications, effectively managed all three cases, providing insights for clinical practice.

To minimize interactions between antimicrobial drugs and immunosuppressants, corticosteroids were used in the initial phase to reduce immune rejection and inflammation. A 2023 review by Silva et al. (18) highlighted the role of ISA in treating invasive fungal infections in solid organ transplant recipients, emphasizing the importance of adjusting tacrolimus and mTOR inhibitor dosages during treatment to mitigate drug interactions.

In cases of ICHs with severe pneumonia from complex infections, rapid pathogen identification is critical. Traditional diagnostic methods, such as smears, cultures, and serological tests, are inefficient and lack specificity. Advanced molecular techniques like mNGS and PCR enable rapid and sensitive identification of pathogens, even for those undetectable by conventional methods (19). In this case series, immediate bronchoscopy and BALF testing using mNGS clearly identified multiple pathogens and contributed to effective therapy.

Subsequent treatment was tailored based on pathogen virulence, disease progression, treatment duration, patient condition, and medication tolerance. All three patients had multi-system dysfunction and limited tolerance to certain drugs, necessitating a phased treatment approach. Priority was given to rapidly progressing pathogens with high mortality rates, such as PJ, *Nocardia*, and CMV. Epidemiological data from Germany (2014–2019) show a rise in PJ incidence from 1,857 to 2,172 cases, with related mortality increasing from 516 to 615 cases (20, 21). Early diagnosis and treatment are necessary for PJP patients because of the high risk of respiratory failure. *Nocardia* pneumonia, which can cause serious complications and life-threatening infections, requires prioritized treatment too (22). CMV pneumonia, which progresses rapidly, can increase the risk of infections from other pathogens like PJP, elevate the risk of disseminated NTM infections, and intensify the severity of COVID-19 infections. Therefore, CMV is also addressed in the initial treatment phase (23–25). In contrast, early *Aspergillus* infections often show no symptoms and progress relatively slowly in hosts without neutropenia, while symptoms usually appear several weeks later (26). NTM has a long treatment period (usually over 12 months), and its progression is relatively slow (27). Three patients are non-neutropenic hosts. Hence, controlling fast-progressing and highly lethal pathogen infections should be the first priority, followed by long-term comprehensive antibiotic treatment for *Aspergillus* and NTM.

Furthermore, it’s significant to select antimicrobial drugs that are not only highly effective but also have minimal side effects. Medications for aspergillosis include polyenes, echinocandins, flucytosine, and triazoles. Notably, triazoles consist of itraconazole, posaconazole, VOR, and ISA. ISA, as the latest generation of triazoles, has several advantages, including fewer adverse reactions, straightforward drug interactions, linear and predictable pharmacokinetics, and no QTc interval prolongation (5). Compared to other triazoles, ISA claims to have an oral and intravenous bioavailability of nearly 98%, which is unaffected by food intake, changes in stomach pH, or mucositis (28, 29). Moreover, ISA has the longest half-life and exhibits high tissue penetration. ISA achieves stable drug concentrations in nearly all tissues, including the brain (which has a blood-brain barrier), within two weeks. Importantly, it doesn’t accumulate in physiological fluids or tissues over time (30). However, lipophilic itraconazole and posaconazole may not adequately reach some organs, such as the prostate, brain, and eyes (31). VOR, a primary treatment for invasive fungal diseases since 2002, has a narrow therapeutic window and complex drug interactions. The interactions arise particularly with drugs commonly used in the ICU, due to VOR’s metabolism involving CYP2C19, CYP2C9, and CYP3A4 (32–34). ISA is a recommended treatment for IPA because of the lowest incidence of side effects among triazoles and its efficacy (35). In 2016, Maertens et al. conducted a Phase III, double-blind, global, multicenter, randomized controlled non-inferiority trial that confirmed the efficacy of ISA in treating IPA to be comparable to that of VOR, with fewer adverse reactions (36, 37). Further analysis by Horn et al. (38) highlighted ISA’s ability to significantly reduce hospitalization duration for patients with moderate to severe renal insufficiency. And ISA is the only triazole that doesn’t require routine therapeutic drug monitoring, according to the Guidelines of the Infectious Diseases Society of America and the European Conference on Infections in Leukemia (39, 40). In 2023, a multicenter observational case study in China reported a 75% remission rate for ISA in the treatment of invasive fungal diseases (41). ISA serves as a safe and effective alternative for patients who are resistant or intolerant to other triazoles (42). Throughout the treatment process, our therapeutic decisions were guided by the 2016 IDSA Clinical Practice Guideline, which recommends a minimum treatment duration of 6–12 weeks for IPA (43). In this case series, ISA demonstrated excellent tolerability and efficacy. In Case 1, VOR (13 days) was replaced with ISA (45 days) due to inadequate drug concentrations caused by rapid metabolism. In Cases 2 (ISA: 66 days) and 3 (VOR: 21 days; ISA: 45 days), ISA’s lower toxicity and improved tolerability made it the preferred choice for younger patients with abnormal liver and renal function. Additionally, first-line antibacterial treatment with SMX-TMP effectively managed PJP in all three cases (44). ISA’s benefits include fewer drug interactions, reduced liver and kidney toxicity, and cost-efficiency, as shown by economic models in the UK, US, and Sweden (45–47). According to our follow-up calls or CT scans (Figures 1I–L, 5F, G), none of these patients experienced a recurrence.

The limitations of our paper include patients discontinued ISA treatment due to financial constraints. Consequently, we lack clinical data on comprehensive treatment regimens for ICHs infected with IA and other pathogens. Further studies are warranted to assess ISA’s long-term efficacy. Additionally, because of the extremely low survival rate of ICHs co-infected with IA and other pathogens, we only report three successful cases. ICHs in our study have different underlying diseases taking various immunosuppressants and steroids. Consequently, it is challenging to summarize and formulate a general guideline on how to adjust the doses of specific immunosuppressants and steroids when treating complex infections of IA and other pathogens. The concurrent use of multi-drug therapies is another constraint of the study. Therefore, more controlled studies are needed to evaluate the efficacy of ISA accurately in the future. While our case series reports suggest ISA’s potential clinical utility, further large-scale, multicenter trials are warranted to validate these observations. Nevertheless, our study addresses the existing gaps in the clinical use of ISA for complex infections. Moreover, our study offers a novel insight into the concurrent administration of ISA with immunosuppressants and corticosteroids. Furthermore, our study contributes to improved survival rates in the treatment of ICHs with complex infections. Collectively, this study highlights ISA as a valuable option for treating ICHs with IPA and complex co-infections.

## Conclusion

This study highlights the challenges and complexities of managing severe pneumonia with IPA and co-infections in ICHs. Prompt pathogen identification using advanced diagnostic methods, such as mNGS, and a phased treatment approach prioritizing rapidly progressing pathogens, were critical for successful outcomes. ISA demonstrated excellent efficacy and tolerability in all three cases, offering a safe and effective alternative to VOR, particularly in patients with compromised hepatic and renal function. The use of ISA minimized drug interactions and adverse effects, ensuring better compatibility with the patients’ underlying conditions and concurrent therapies. These findings underscore the importance of individualized, stepwise treatment strategies in ICHs and support ISA as a valuable option for the management of IPA and complex co-infections, paving the way for improved clinical outcomes in this high-risk population.

## Data Availability

The original data presented in this study are included in this article/supplementary material, further inquiries can be directed to the corresponding authors.
